# Supplementary material for: Altered mRNA and Protein Expression of Monocarboxylate Transporter MCT1 in the Cerebral Cortex and Cerebellum of Prion Protein Knockout Mice
Source: Int J Mol Sci. 2021 Feb 4;22(4):1566. doi: 10.3390/ijms22041566 (PMC7913939; doi:10.3390/ijms22041566)
Supplement: Supplementary file 1 [file ijms-22-01566-s001.zip › ijms-1091057-supp-revise.pdf]

Supplemental Table S1: Overview on mice behavioral deficits in Prnp-/- mice during aging (adapted from Schmitz et al., 2014).

Behavior	3 Months WT	3 Months Prnp-/-	9 Months WT	9 Months Prnp-/-
Associative learning	=	=	=	↓↓
Anxiety	=	=	=	↓↓
Curiosity and memory	=	=	=	↓↓
Motor skills	=	=	↓	↓

"=" = no significant differences compared to 3 months WT; " ↓ " = Minor deficits compared to 3 months WT; " ↓ ↓ " = Moderate deficits compared to 3 months WT; " ↓ ↓ ↓ " = Severe deficits compared to 3 months WT.

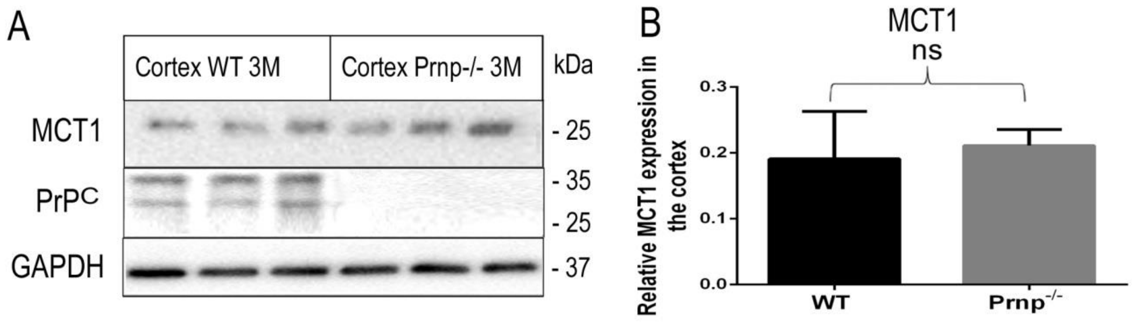

Supplemental Figure S1 (A,B). Western blot and densitometric analysis of MCT1 and PrP^c expression in the cortex of 3-month-old WT and Prnp$^{-/-}$ mice. Homogenates prepared from cortex, were examined for MCT1 and PrP^c expression by Western blotting. MCT1 expression was not significantly changed in 3-months old WT as compared to Prnp$^{-/-}$ mice.
