# Supplementary figures and images for: Altered mRNA and Protein Expression of Monocarboxylate Transporter MCT1 in the Cerebral Cortex and Cerebellum of Prion Protein Knockout Mice
Source: Int J Mol Sci. 2021 Feb 4;22(4):1566. doi: 10.3390/ijms22041566 (PMC7913939; doi:10.3390/ijms22041566)

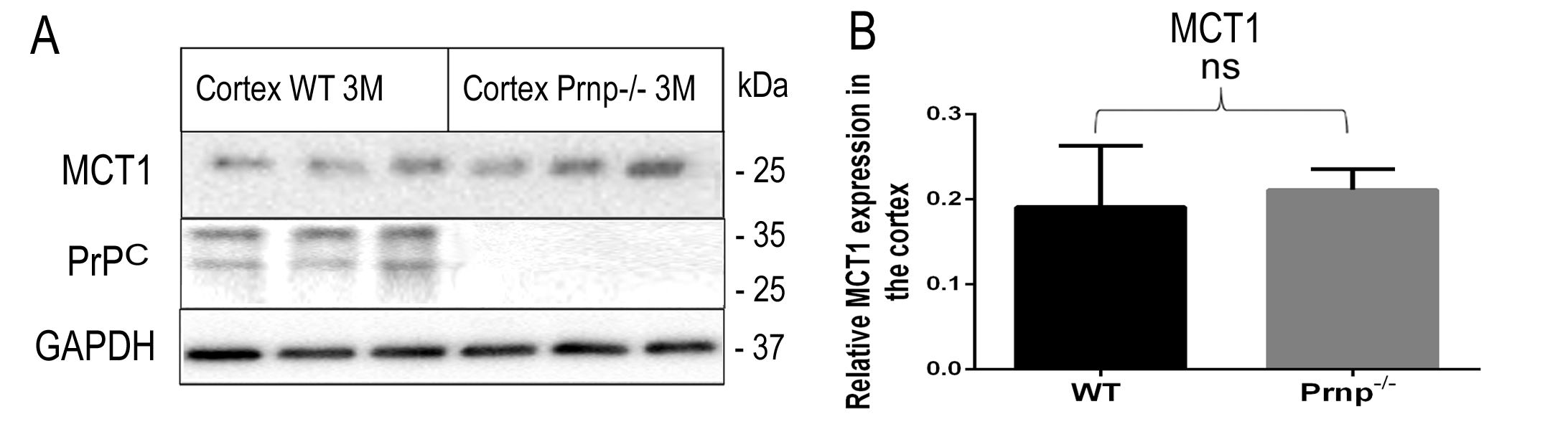

Supplement: Supplementary file 1 [file ijms-22-01566-s001.zip › ijms-1091057-supp-revise.tif]
